# Etiological and risk factors in recurrent febrile seizures: Insights through EEG analysis

**DOI:** 10.5339/qmj.2023.32

**Published:** 2023-12-26

**Authors:** Umesh Babu Kuruva, Vasudev Kompally, Subhan Basha Bukkapatnam, Prathap Gudi, Ramesh Kandimalla

**Affiliations:** ^1^Department of Pediatrics, Kakatiya Medical College, Warangal, Telangana, India; ^2^Department of Biochemistry, Kakatiya Medical College, Warangal, Telangana, India Email: ramesh.kandimalla@gmail.com ORCID iD: 0000-0002-3313-4393

**Keywords:** Febrile seizures, children, EEG, recurrence, risk factors, sodium levels

## Abstract

**Background:**

Febrile seizures, convulsive episodes in young children during febrile illnesses, are a significant concern due to their potential for recurrence and associated uncertainties. This study investigated the causes and risks associated with recurrent febrile seizures and the critical role of electroencephalogram (EEG) in their accurate diagnosis.

**Methods:**

Following Institutional Review Board approval and going through the consenting process with parents, this study gathered the clinical features and EEG recordings of children admitted with febrile seizures in the Department of Pediatrics, Mahatma Gandhi Memorial Hospital, Kakatiya Medical College, Warangal, Telangana, India. Descriptive statistics, including mean, standard deviation (SD), frequencies, and percentages, were computed to understand the data comprehensively. The Chi-Square test was employed to analyze the association between variables, with a significance level of 0.05, ensuring reliable and trustworthy findings.

**Results:**

Out of 42 children studied, 28 (66.67%) presented with simple febrile seizures, with the mean time of occurrence of seizures from the onset of fever being 7.85 hours. Abnormal EEG was seen in 50% of children with complex febrile seizures and 16% with simple febrile seizures. Generalized epileptiform discharges were the most common epileptic activity observed. Low sodium levels had a significant relationship with febrile seizures in the analysis.

**Conclusions:**

This study emphasizes the importance of EEG in diagnosing febrile seizures, particularly in complex cases. Our findings suggest that low sodium levels may be a significant risk factor for febrile seizures. Further research is necessary to identify other preventable risk factors to reduce the burden of the medical condition.

## Introduction

Febrile seizures (FS), as defined by the International League Against Epilepsy (ILAE), are characterized by seizures that occur in children between the ages of 6 and 60 months, accompanied by a body temperature of 37.7°C (100.0°Fahrenheit) or higher. It is crucial to note that these seizures should not be attributed to any underlying central nervous system infection or metabolic imbalance, and they must occur in the absence of a history of prior afebrile seizures.^1^

The hallmark feature of FS lies in their connection with a feverish ailment, during which the body temperature surpasses 37.7°C. Notably, the temperature rise may even occur after the seizure episode. FS is a common reason for pediatric emergency department admissions, affecting around 2-5% of young children.^2,3^

In most cases, FS are considered benign and typically do not require specific treatment or extensive diagnostic workup. They are known to have a favorable prognosis, with the seizures ceasing spontaneously and most children not experiencing long-term neurological consequences.^4,5^ However, despite this understanding, there remains to be considerable variation in the management approaches employed for febrile seizures, leading to a need for further investigation and consensus in clinical practice.

Interestingly, while research on afebrile seizures has received considerable attention, limited studies have been conducted in developing countries to explore the causes and outcomes of seizures, specifically in children with febrile episodes.^6-8^ This research gap motivates the current study, which aims to bridge this knowledge deficit by thoroughly investigating the various etiological factors and risk elements associated with the recurrence of FS.^8^ Additionally, the study seeks to shed light on the crucial role of electroencephalography (EEG) in accurately diagnosing febrile seizures. By exploring these aspects, the research team aims to contribute to the existing literature and provide valuable insights that can inform clinical practice, improve patient management, and enhance outcomes for children experiencing recurrent febrile seizures.^8^ This research aims to identify preventable risk factors to reduce the burden of medical conditions.

## Methods

This prospective observational study was conducted in the “Department of Pediatrics at Public Service (Mahatma Gandhi Memorial) Hospital,” Kakatiya Medical College, Warangal, India, from November 2019 to October 2021. The stratified sampling technique was utilized in this observational study. After obtaining informed assent from parents, children between the ages of 6 months and five years with FS were included in the study. A proforma capturing information on the type of seizures, family history, past episodes, cause of the febrile fit, sodium levels upon admission, and EEG results were completed. The EEG recording for each child took place one week after the seizure episode. The sample size was determined based on the guidelines outlined in “How to calculate sample size for different study designs in medical research?”^9^ and “How do you calculate sample size for observational and experimental nursing research studies?”.^10^ Based on prevalence, the power of the study yielded 150, and the same number was included in the study. The study received ethical approval from the Kakatiya Medical College Ethics Committee, Warangal, India (KMC/IEC/Paed/2019-008).

### Statistical Analysis

The collected data was meticulously entered into Microsoft Excel (Windows 7; Version 2007) to ensure the accuracy of the findings. Analyses of the data were then conducted using the latest Statistical Package for Social Sciences (SPSS) for Windows software (IBM Corp. Released 2020. IBM SPSS Statistics for Windows, Version 27.0. Armonk, NY: IBM Corp), which allowed for complex statistical calculations to be performed with ease. Descriptive statistics such as mean and standard deviation (SD) for continuous variables and frequencies and percentages for categorical variables were carefully calculated to provide an accurate and comprehensive understanding of the data. The association between variables was analyzed using the Chi-Square test for categorical variables, which helped determine any significant associations between the variables under study. The significance level was set at a low value of 0.05, which helped to ensure that the study’s findings were reliable and trustworthy.

## Results

**Figure 1.**
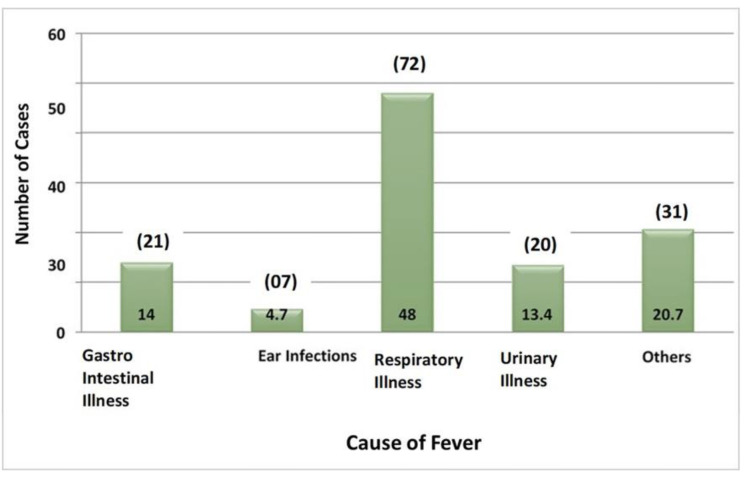
Causes of fever among the pediatric patient sample in this study.

This study involved an analysis of 150 children who were evaluated based on their duration of presentation with seizures from the onset of fever, family history of FS and seizure disorder, anemia levels, temperature at presentation, C-reactive protein (CRP) test results, and sodium levels (Tables 1 and 2). Out of the children studied, most arrived at the hospital within 6 hours of the onset of fever. A significant portion came within 6 to 12 hours, and a smaller percentage arrived after 12 hours of fever onset. The average time between fever onset and seizure presentation was 7.85 hours. A portion of the study group had a family history of FS, and a smaller number had a history of seizure disorder (Table 1; Figure 1).

**Figure 2.**
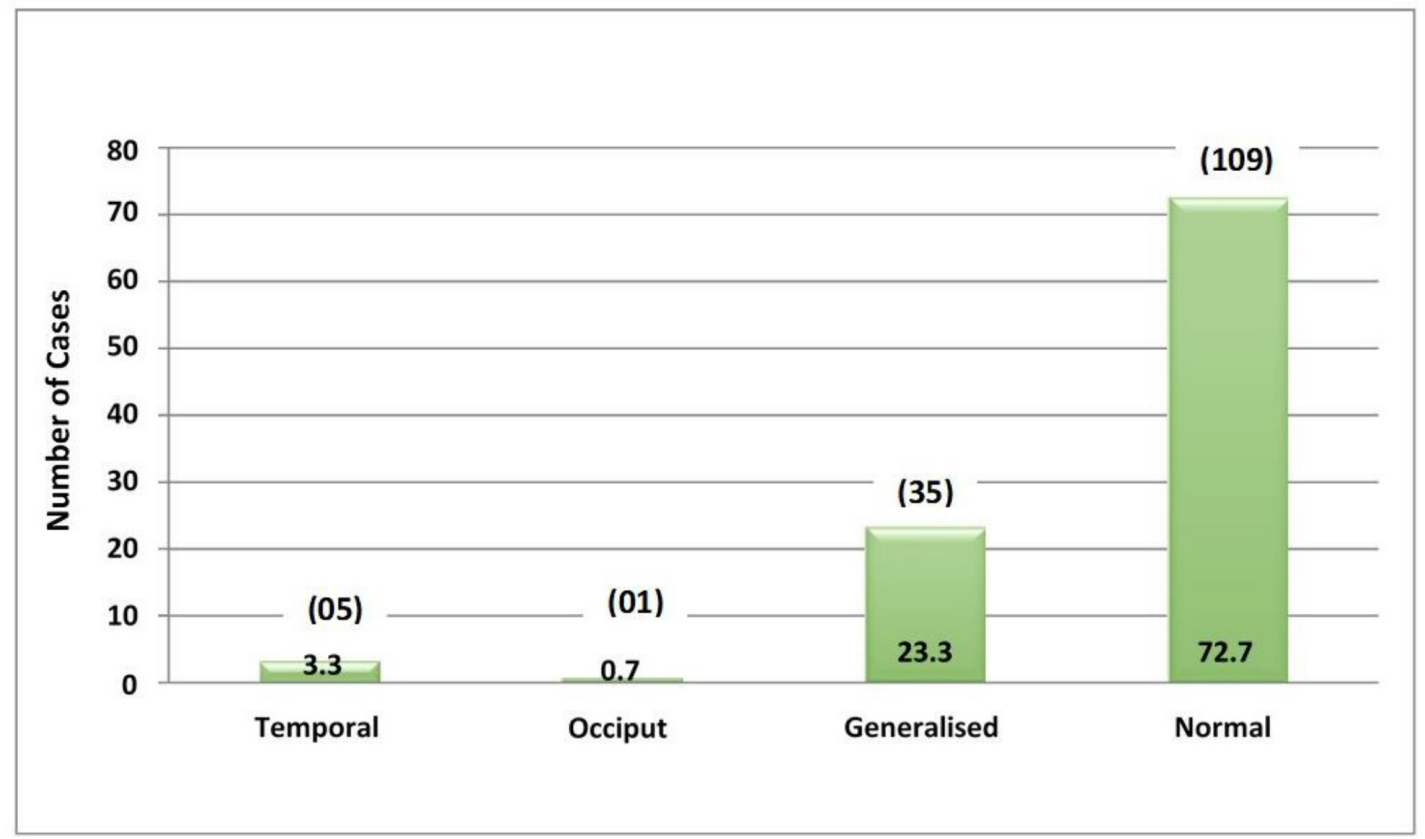
Results of EEG test conducted on pediatric patients.

Magnetic Resonance Imaging (MRI) was performed for a subgroup of 9 children, which encompassed a history of recurrent FS, delayed onset of their initial FS, and the presence of focal seizures, with results indicating temporal lobe sclerosis in 2 cases. Within the larger group of 150 children, a range of anemia severity was observed: some displayed no anemia, others had mild or moderate anemia, and a few presented with severe anemia (Table 2; Figures 2 and 5).

The analysis of initial body temperatures revealed that children had varying readings, ranging from 37.2 to 39.4 degrees Celsius (99 to 103 degrees Fahrenheit). A positive CRP test was observed in cases across different temperature ranges, with the Chi-square test indicating a highly significant p-value of less than 0.001.

**Figure 3.**
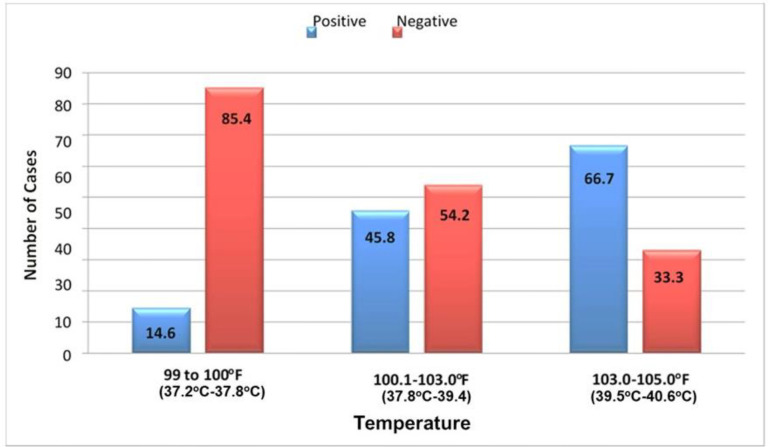
Temperature ranges of positive and negative CRP test results.

In the assessment of initial body temperatures, a spectrum of temperature levels was noted among the children. Among 48 children with temperatures ranging from 37.2 to 37.8°C (99 to 100°F), 7 displayed positive CRP results. Similarly, in a subset of 96 children with temperatures spanning 37.8 to 39.4°C (100.1 to 103.0°F), 44 exhibited positive CRP results. Furthermore, only three children registered temperatures surpassing 39.4°C (103°F); of this group, two tested positive for CRP. The statistical significance of these findings was established by applying the Chi-square test, revealing a p-value of less than 0.001. Notably, a comparison was drawn between the occurrence of CRP-positive cases in the lower (37.2 to 37.8°C) and higher (37.8 to 39.4°C) temperature ranges (Table 2; Figure 3).

**Figure 4.**
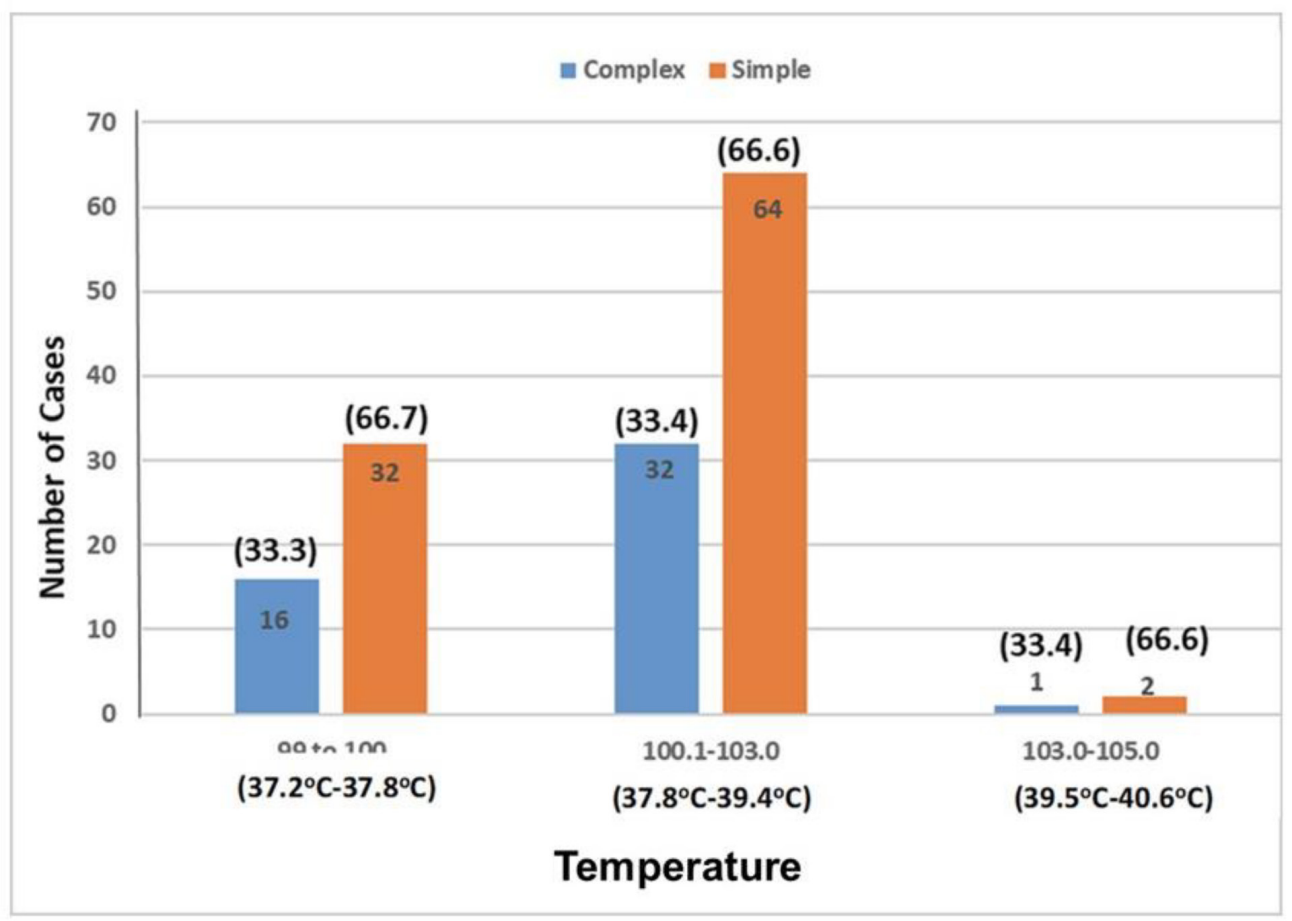
Temperature ranges of simple and complex FS cases.

**Figure 5.**
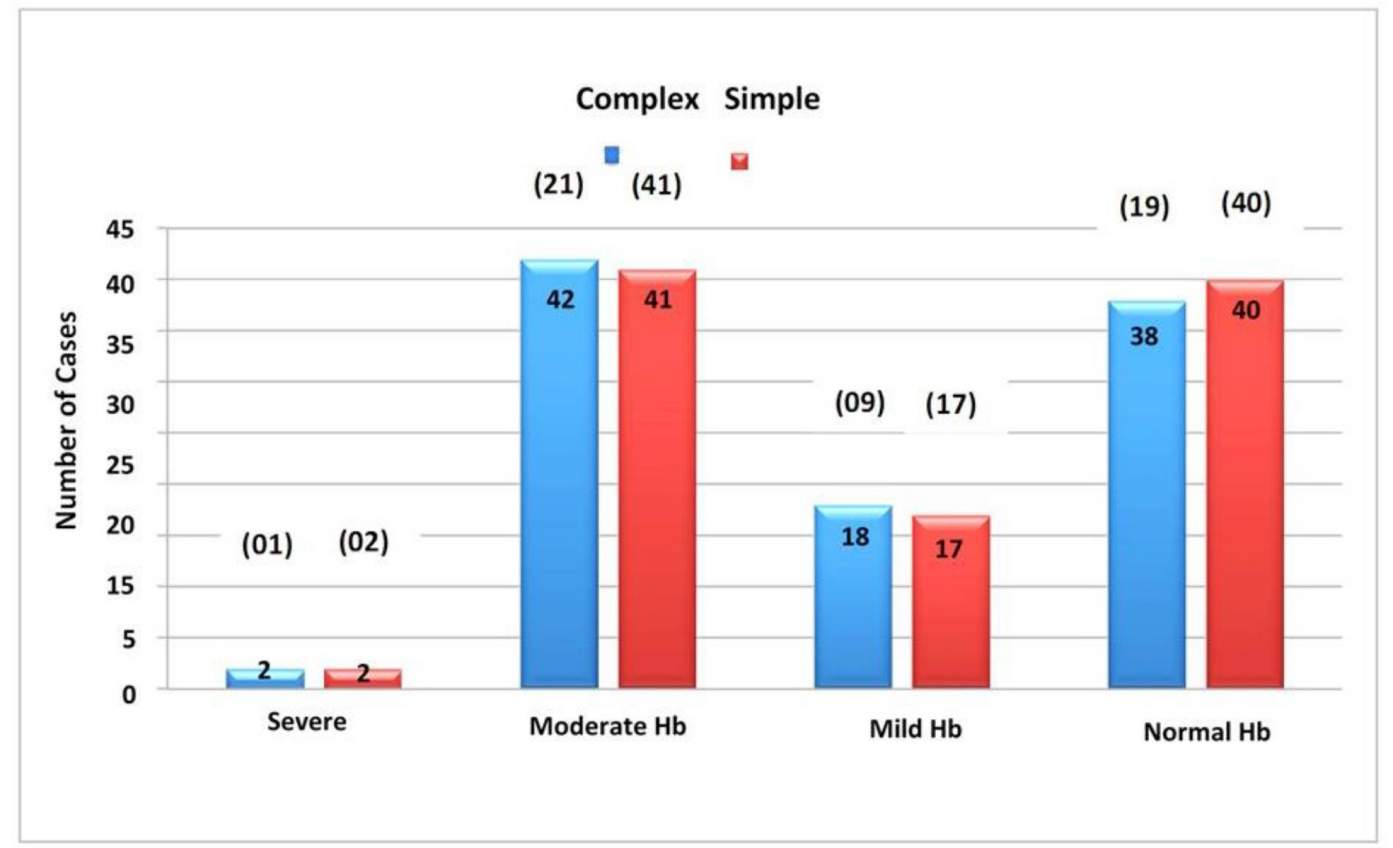
Signs of anemia in children with simple and complex FS cases.

Figure 4 depicts the relationship between Seizure Type and Temperature. In the temperature range of 37.2-37.8°C (99-100°F), 48 children were observed, while 96 children fell within the 37.8-39.4°C (100.1-103°F) range. A subset of 3 children exhibited temperatures above 39.4°C (103°F). Among those experiencing complex FS, 16 showed temperatures of 37.2-37.8°C (99-100°F), 32 had temperatures of 37.8-39.4°C (100.1-103°F), and 1 had temperatures exceeding 39.4°C (103°F). For children with simple FS, 32 had temperatures of 37.2-37.8°C (99-100°F), 64 had temperatures of 37.8-39.4°C (100.1-103°F), and 2 had temperatures surpassing 39.4°C (103°F). Figure 5 demonstrates the relationship between seizure type and hemoglobin (Hb) levels. Different anemia levels were observed for children with complex FS, while a similar pattern was noted among those with simple FS. The statistical analysis using the Chi-Square test resulted in a p-value of 0.996, indicating the absence of a statistically significant correlation between Seizure Type and Hb levels.

**Figure 6.**
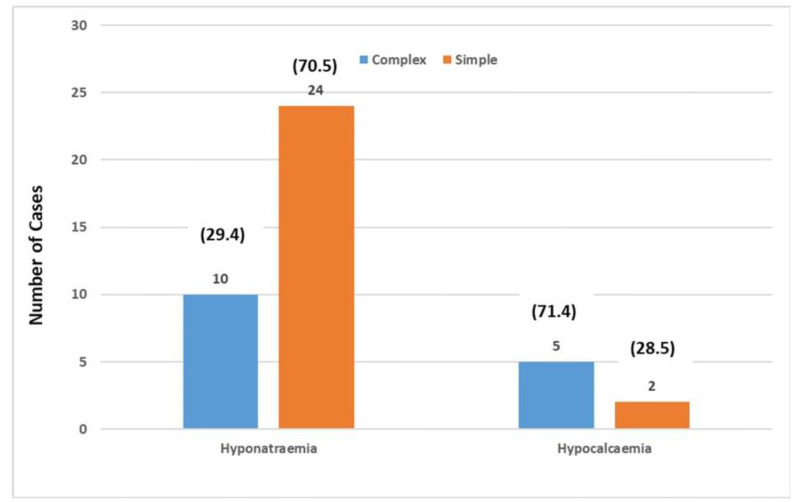
Hyponatremia outcomes in simple and complex FS cases.

A distinct pattern emerged within the cohort of children diagnosed with hyponatremia: 10 exhibited complex FS, while 24 experienced simple FS. The relationship between hyponatremia and seizure type was found to be highly significant, with a p-value of less than 0.0016. Moreover, among children with simple FS, 84% had average EEG results, while 16% showed abnormalities. In contrast, 50% displayed EEG abnormalities for children with complex FS. Notably, among positive EEG records, generalized abnormalities were the most prevalent (23.3%), followed by temporal activity anomalies (3.3%) and occipital activity anomalies (0.7%) (Figures 2 and 6).

**Figure 7.**
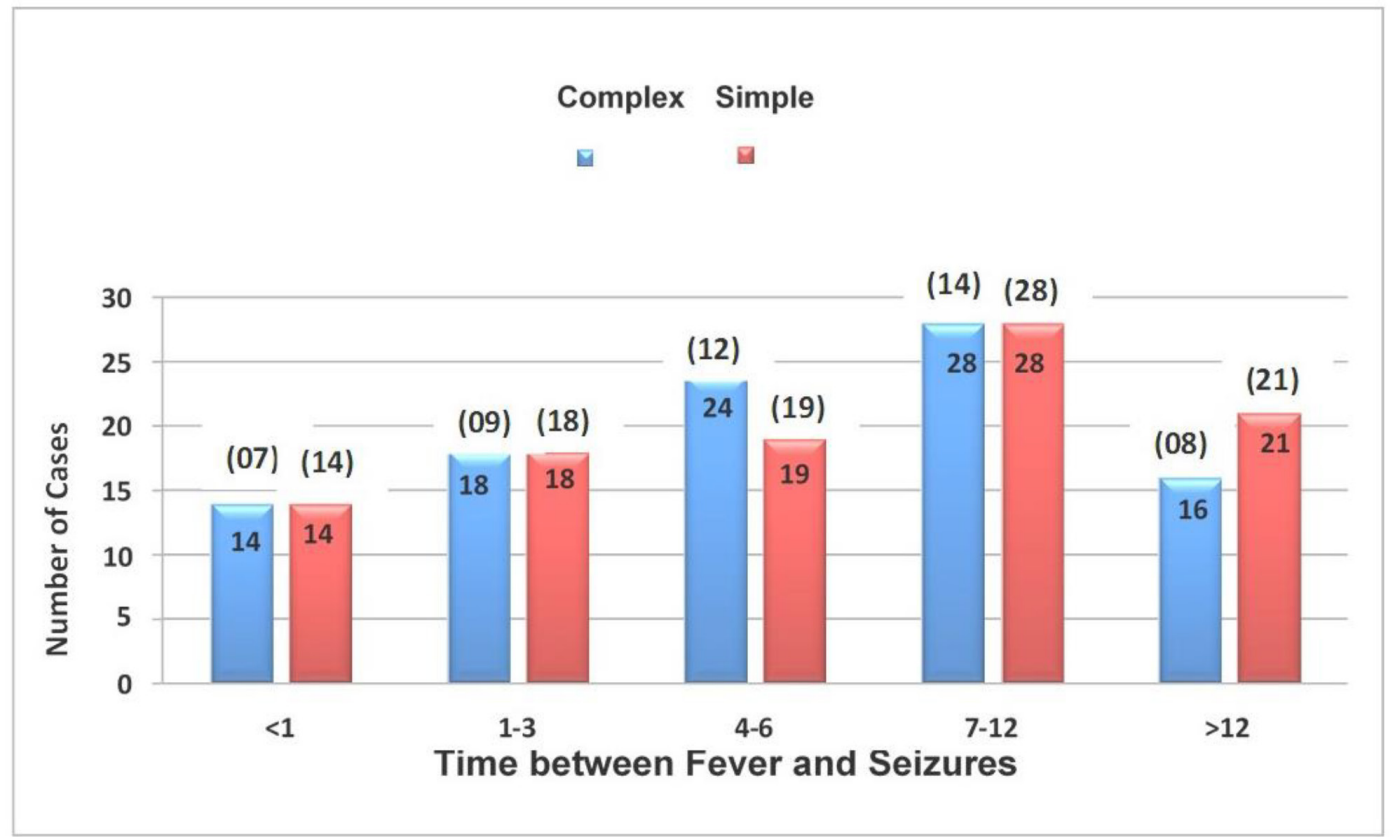
Time between fever and seizures in simple and complex FS cases.

**Figure 8.**
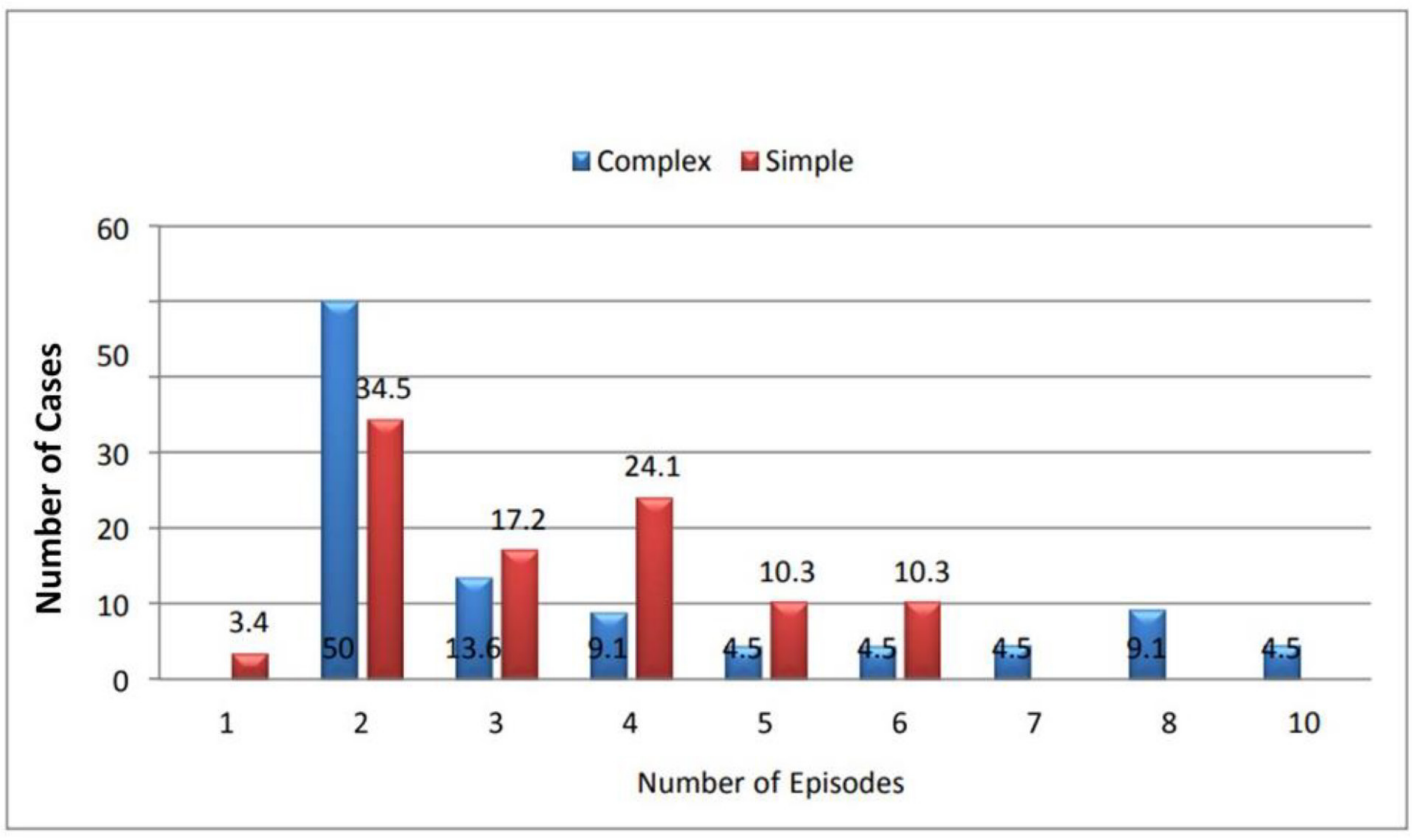
Number of seizure episodes in simple and complex FS cases.

Figure 7 illustrates the relationship between Seizure Type and Time Interval between Fever and Seizures for simple and complex FS. Despite varying timings within each group, statistical analysis did not find a significant association. The mean duration between fever onset and seizures was similar for both seizure types. Figure 8 explores Seizure Type and Number of Episodes, revealing comparable distributions for both groups. The average number of episodes was marginally higher for complex FS, but statistical analysis did not yield significant results.

## Discussion

This prospective observational study examined children with FS’s clinical, laboratory, and EEG characteristics.^3^ FS occurs when a child’s body temperature rises due to an infection outside the central nervous system (CNS). In this study, the most common causes of FS were viral infections, malaria, pneumonia, otitis media, measles, urinary tract infections, septicemia, and pharyngotonsillitis. Vaccination with the diphtheria, tetanus, and pertussis (DPT) vaccine can also cause FS.^4-7^

While the exact cause of FS is unclear, it is believed to be a combination of factors, including an immature brain, fever, and genetic predisposition.^8^ Children who are susceptible to FS have limited capacity to increase cellular energy metabolism at elevated temperatures, which can result in anoxic insult to the brain, leading to convulsions.^8^ The rise of temperature and the actual peak are vital factors.

The concept of our study results is in line with Gallentine et al. study^22^ findings, which showed that interleukins (IL)-8 and Epidermal growth factor (EGF) levels were significantly elevated after febrile status epilepticus (FSE) compared to the control group. Although IL-1β levels showed a higher trend and IL-1RA showed a lower trend following FSE, the differences did not reach statistical significance.^22^ Children with FSE had significantly lower ratios of IL-1RA/IL-1β and IL-1RA/IL-8. While individual cytokine levels did not correlate with FSE, lower ratios of IL-1RA/IL-1β, IL-1RA/IL-6, and IL-1RA/IL-8 were all associated with FSE.^22^ In children with T2 hippocampal hyperintensity on MRI after FSE, IL-6, and IL-8 levels were significantly higher, and ratios of IL-1RA/IL-6 and IL-1RA/IL-8 were substantially lower than those without hippocampal abnormalities. Individual cytokine levels and IL-1RA/IL-1β or IL-1RA/IL-8 ratios did not predict MRI changes. However, a lower IL-1RA/IL-6 ratio strongly predicted the occurrence of hippocampal T2 hyperintensity after FSE (OR 21.5, 95% CI: 1.17–393).^22^ It also provides evidence of the involvement of the IL-1 cytokine system, IL-6, and IL-8 in FSE among children. The most significant finding is the identification of the IL-1RA/IL-6 ratio as a potential biomarker for acute hippocampal injury following FSE.^22^ The study specifically examined the relationship between temperature and CRP with febrile seizures. Dysregulation between proinflammatory cytokines (IL1, IL-6, IL-8) and anti-inflammatory cytokines has been associated with FSE. The study also found that hyponatremia was significantly associated with febrile seizures.^22^ In the current study, among the examined children, 14.6% [7 out of 48 of the group-37.22°C to 37.78°C (99 to 100 Fahrenheit)] tested positive for CRP. In a subgroup of 96 children with temperatures ranging from 37.83°C to 39.44°C (100.1 to 103.0°Fahrenheit), 45.8% (44 children) tested positive for CRP. Interestingly, out of the three children with temperatures above 39.44°C (103°Fahrenheit), 66.7% (2 children) tested positive for CRP. The statistical analysis using a Chi-square test yielded a highly significant p-value of <0.001, indicating the strong association between temperature range and CRP positivity. Specifically, when comparing the two groups of CRP-positive children, the lower temperature range had 14.6% (7 children), while the higher temperature range had a significantly higher percentage of 45.8% (44 children). Previous studies^22^ and our findings suggest that low sodium levels may be a significant risk factor for febrile seizures.

One-third of children with FS experience a recurrence, and 10% have three or more recurrences. EEG is usually only performed in certain cases where epilepsy is suspected, such as in cases of recurrent FS, complex FS, fever within 1 hour of an FS, family history of epilepsy, and neurodevelopmental abnormalities.^1,4-8^ Computed Tomography (CT) or MRI is not recommended after a first simple FS, but they may be necessary in cases of FSE.^8,22^

The study found that 84% of children with simple FS had a normal EEG, while 50% of children with complex FS had abnormal EEG findings. In children with FSE, focal EEG slowing over the temporal lobe increases the chance of medial sclerosis on follow-up.18 Additionally, 10% of children with FSE had unilateral or bilateral swelling of the hippocampus, with subsequent hippocampal atrophy in 71% of those with acute findings.^14,18,19^

The current study found a significant connection between FS and hyponatremia, consistent with previous research conducted by Javeri et al.^20^ Additionally, the present study showed that 35.3% of children who tested positive for CRP had a high likelihood of having an infectious cause of fever. We also observed that the chance of infection increased as temperature rose, similar to Javeri et al.’s findings.^20^ However, their study had a slightly higher percentage (45%) of children with an infectious cause of fever. Moreover, in our research, a large majority (84%) of children with simple FS had a normal EEG, which was a highly significant finding. On the other hand, half of the children with complex FS had an abnormal EEG, which aligned with the results of a study by Jeong et al.,^21^ where among the 131 children included in the analysis, 103 had simple FS, while 28 had complex FS. EEG abnormalities were observed in 41 children (31%). Although EEG abnormalities were more common in children with complex FS than those with simple FS (43% vs. 28%), the difference was insignificant. Logistic regression analysis revealed that having multiple seizures within 24 hours significantly predicted abnormal EEG results (odds ratio: 2.98; 95% confidence interval: 1.0 to 88; P=0.048). However, the frequency of FS recurrence did not significantly differ between the normal (31%) and abnormal (23%) EEG groups.

In addition, one recent study found that the chance of infection increased with the temperature rise, and almost half of the children had respiratory illness associated with the fever. This finding highlights the importance of monitoring respiratory symptoms in children with fever, as they may be at a higher risk of developing FS.^23^

Recent research has also focused on the potential long-term effects of FS on cognitive development;^24^ school-age children with complex FS showed normal overall intelligence but had lower executive functioning. Children with complex FS exhibited attentional problems, anxiety/depression symptoms, and hyperactivity. The study suggests that while infants with complex FS appear to develop normally in the short term, challenges in executive function and cognitive abilities can emerge in school-age children with a history of FS. Studies have suggested that children who experience FS may be at an increased risk of developing learning and behavioral problems later in life.^25-27^

Overall, this study sheds light on the characteristics and potential causes of FS in children and the role of EEG in diagnosing and managing FS.

## Conclusions

This prospective observational study aimed to investigate the occurrence of FS in 150 children. The results showed that the mean time of seizure onset from the start of fever was 7.85 hours, indicating that febrile seizures are more likely to occur within the first few hours. Interestingly, the study found no statistically significant difference in the incidence of FS, or the type of FS based on temperature differences.

Furthermore, the study observed no statistically significant difference in the recurrences between children with simple FS and those with complex FS. However, it was found that almost one-third of the children had a family history of FS, suggesting that genetics may play a role in the development of this condition.

Therefore, monitoring and providing appropriate care for children with FS is essential to mitigate these potential long-term consequences. Moreover, the research has also identified specific genetic mutations associated with FS, which may help to explain why some children are more susceptible than others. Advances in brain imaging techniques have also allowed researchers to understand the neurological basis of febrile seizures better, providing new insights for future treatments and interventions.

Our study showed no difference between the anemia in children presenting with simple and FS. Complex FS has an increased propensity to progress to an epileptiform disorder when compared to simple FS.

## Author Contributions

The U.K. conceived the concept of the article and structured its written format. V.K. and R.K. designed the article and participated in the study discussion. S.B.B. contributed to the design of the article format style, data analysis, writing, and editing. P.G., S.B.B and R.K. were involved in the final editing and proofreading of the article and revision. Finally, all the authors approved the final version of the article.

## Declaration of Conflict of Interest

None.

## Funding

None.

## Acknowledgement

All authors acknowledged the patients who participated in the study conducted at Kakatiya Medical College-MGM Hospital in Warangal, Telangana, India.

## Figures and Tables

**Table 1.** Clinical data of the study group.

	Number of Patients	Percentage (%)
**Age in Months**		
6-12	42	28.0
13-24	56	37.3
>24	52	34.7
**Gender**		
Girls	49	32.7
Boys	101	67.3
**Age of onset**		
<1 year	78	52.0
1-2 year	46	30.7
>2 years	26	17.3
**Family history**		
Febrile Seizure	42	28.0
Epilepsy	12	8.0
No	96	64.0
**Temperature**		
37°C-37.8°C (98.6°F -100°F)	51	34.0
37.8°C-39.4°C (100.1°F103.0°F)	96	64.0
39.4°C-40.6°C (103.0°F-105.0°F)	3	2.0
**Seizure type**		
Simple FS	100	66.7
Complex FS	50	33.3
**Fever – Seizure time**		
<6 hr	79	53.0
6-12 hr	42	28.0
>12 hr	29	19.0
**Mean duration 7.85 hr**		
**Previous Seizure episodes**		
1	1	0.7
2	21	14.0
3	8	5.2
4	9	6.0
5	4	2.7
6	4	2.7
7	1	0.7
8	2	1.3
10	1	0.7
** 0**	**99**	**66**

**Table 2.** Data about associated symptoms.

Associated Symptoms	Number of Patients	Percentage (%)
**Symptoms**		
Cough/Cold	64	42.7
Only fever	26	17.3
Loose stools	21	14.0
Burning/Crying during Micturition	19	12.7
Vomiting	19	12.7
Ear Pain/Discharge	7	4.7
Cough	6	4.0
Cold	1	0.7
Facial Puffiness	1	0.7
Trauma Over Leg	1	0.7
Rash	1	0.7
**Anemia**		
No	59	39.0
Mild	26	18.0
Moderate	62	41.0
Severe	3	2.0
**CRP**		
Positive	53	35.3
Negative	97	64.7
**CRP vs. Temperature**		
**Temperature**	**CRP Positive**	**CRP Negative**
37.2°C-37.8°C (99°F-100°F)	7 (14.6)	41 (85.4)
37.8°C-39.4°C(100.1°F-103.0°F)	44 (45.8)	52 (54.2)
39.5°C-40.6°C (103.1°F-105.0°F)	2 (66.7)	1 (33.3)
CHI Square Test	P-Value	<0.0001
**Electrolytes vs. Seizure Type**		
Electrolytes	**Simple FS**	**Complex FS**
Hyponatremia	10 (29.4)	24 (70.5)
Hypocalcemia	5 (71.4)	2 (28.5)
Hyponatremia P-Value 0.0019
**EEG vs. Seizure Type**		
EEG	**Simple FS**	**Complex FS**
Normal	84	25
Abnormal	16	25
Uncorrected Chi-Square test	p-value	<0.0001
**Type of EEG Discharges**		
Temporal	5	3.3
Occiput	1	0.7
Generalized	35	23.3

*Note*: Electrolytes were measured in mmol/L.
